# Utility of Preoperative Anesthesia Clinic Videoendoscopy for Airway Management Planning

**DOI:** 10.5812/aapm.19776

**Published:** 2014-09-01

**Authors:** Peter J Kallio, Ana E Cox, Paul S Pagel

**Affiliations:** 1 Anesthesia Service, the Clement J. Zablocki Veterans Affairs Medical Center, Milwaukee, Wisconsin, USA

**Keywords:** Laryngoscopy, Patient Safety, Endotracheal Intubation, Tracheostomy, Videoendoscopy, Preoperative Anesthesia Clinic, Difficult Airway, Laryngeal Carcinoma

## Abstract

**Introduction:**

The authors performed videolaryngoscopy during the preoperative anesthesia clinic evaluation of a patient with chronic dyspnea, stridor, and a previous hemilaryngectomy scheduled to undergo a series of orthopedic surgery procedures for an infected knee arthroplasty. The findings proved crucial for determining airway management.

**Case Presentation:**

A 68-year-old man presented to the preoperative anesthesia clinic for work-up before anticipated removal of infected total knee arthroplasty hardware, placement of antibiotic spacers, incision and drainage procedures, and revision arthroplasty. The patient had previously undergone a hemilaryngectomy and tracheostomy (now closed) for squamous cell carcinoma of the right true vocal cord. The patient described chronic dyspnea with minimal exertion. Inspiratory and expiratory wheezes and intermittent inspiratory stridor were present. A transnasal videolaryngoscopy examination was performed using topical anesthesia and demonstrated significant supraglottic scarring, a narrowed glottis, and subglottic stenosis. A computed tomography study confirmed the presence of tracheomalacia with subglottic stenosis. A permanent tracheostomy was performed to establish a definitive airway before the knee arthroplasty was removed.

**Conclusions:**

The case illustrates that transnasal videolaryngoscopy conducted in the preoperative anesthesia clinic is capable of providing key information to guide airway management in patients with significant upper airway pathology.

## 1. Introduction

Transnasal flexible fiberoptic or videoendoscopy is a standard tool used in otolaryngology and speech pathology clinics for dynamic evaluation of the upper airway including the distal oropharynx, glottis, and proximal trachea in adults (1-5) and children (6). The procedure is most often performed using topical anaesthesia alone (2, 7) is well tolerated by the vast majority of patients without the need for sedation (4, 6), and yields important information about airway pathology and its treatment (8, 9). Anesthesiologists are trained to use fiberoptic bronchoscopy to facilitate endotracheal intubation in the conscious or anesthetized patient with a suspected or known difficult airway (10-13), but the use flexible videoendoscopy for airway assessment has only been recently described immediately before surgery in conjunction with attempts to reduce unnecessary awake intubation (14). The authors have performed flexible videoendoscopy in their preoperative anesthesia clinic on selected patients for several years, and the following case illustrates the utility of this technique for airway management planning. The authors conducted their examination in a patient with dyspnea on exertion, stridor, and a previous hemilaryngectomy scheduled to undergo a series of orthopedic surgery procedures for treatment of an infected knee arthroplasty. The videoendoscopy findings proved to be crucial for determining this patient’s airway management. The patient provided his written consent for publication of this report.

## 2. Case Presentation

**Figure 1.**
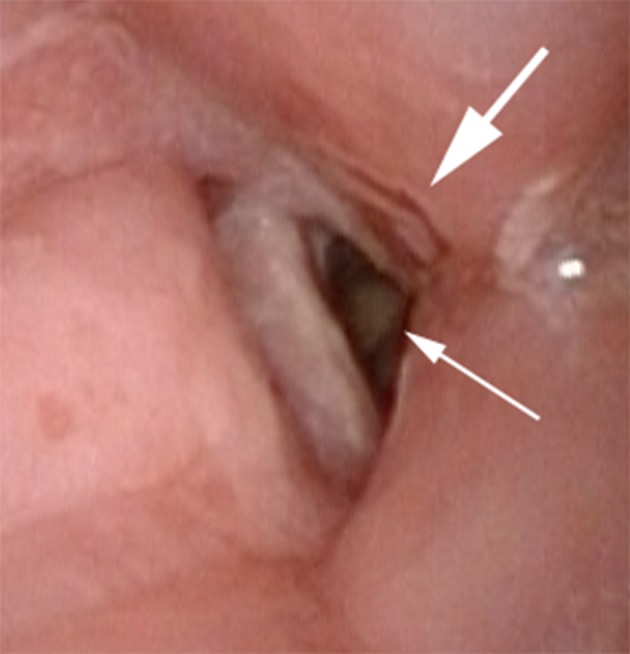
Photograph Obtained During Transnasal Videoendoscopy Demonstrating Absent Right Vocal Cord, Narrowed Glottic Opening, Supraglottic Scarring (Large Arrow), and Subglottic Stenosis (Small Arrow)

A 68-year-old, 179 cm, 108 kg man presented to the authors’ preoperative anesthesia clinic for evaluation before anticipated removal of infected total knee arthroplasty hardware, placement of antibiotic spacers, incision and drainage procedures, and revision arthroplasty. The patient had undergone a right total knee arthroplasty for osteoarthritis eight years before the current evaluation, but he subsequently developed a draining sinus tract in the operative joint consistent with a chronic infection that had not responded to conservative treatment. The patient’s past medical history was notable for obstructive sleep apnea, tobacco abuse, moderate to severe chronic obstructive pulmonary disease (documented with pulmonary function tests; responsive to inhaled bronchodilators), and squamous cell carcinoma of the right true vocal cord (stage T2N0) for which he had previously undergone a right vertical hemilaryngectomy nine years before current evaluation. A tracheostomy that was performed as part of the hemilaryngectomy had been allowed to spontaneously close as he recovered from the operation. The patient was not treated with head and neck radiation therapy nor did he receive adjuvant chemotherapy. The patient reported that he was chronically short of breath and had been intermittently since the tracheostomy closed. He was unable to tolerate continuous positive airway pressure equipment for treatment of his obstructive sleep apnea. He described dyspnea on exertion with minimal exercise. Indeed, he was noticeably short of breath after walking from the waiting room to the clinic office (a distance of less than 50 feet). Reduced breath sounds with inspiratory and expiratory wheezes were heard during auscultation of his lungs. Intermittent inspiratory stridor was also present. The patient’s voice was coarse and soft in character. A Mallampati class 3 upper airway was present, the thyromental distance was approximately 5 cm, and cervical osteoarthritis limited the patient’s ability to extend his neck. These findings suggested that endotracheal intubation may be somewhat more difficult using direct laryngoscopy, but based on this standard assessment, the authors felt that the patient’s airway could be easily secured using a videolaryngoscope. Nevertheless, the previous hemilaryngectomy, closed tracheostomy, obstructive sleep apnea, and mild inspiratory stridor, prompted the authors to perform a transnasal videoendoscopy examination of the upper airway. Topical local anesthesia [consisting of 4% lidocaine mixed with phenylephrine (200 mcg)] was applied into the right nares and upper oropharynx using an atomizer with the patient in sitting position. A videoendoscope (Olympus ENF-VH, Tokyo, Japan) was then gently passed through the right nares and advanced through the oropharynx to a position immediately above the glottis. Absence of the right arytenoid complex was noted consistent with the previous hemilaryngectomy (Figure 1). The left vocal cord moved normally with phonation, but supraglottic scarring narrowed the glottic opening. Subglottic airway narrowing was also observed beyond the glottic opening (Figure 1); the authors did not attempt to further examine the proximal trachea as a result. A large amount of redundant tissue was also noted in the lower oropharynx that caused partial airway obstruction with the patient breathing spontaneously. This latter finding was consistent with the established diagnosis of obstructive sleep apnea. The videoendoscope was removed after the examination was completed. The patient tolerated the procedure well.

**Figure 2.**
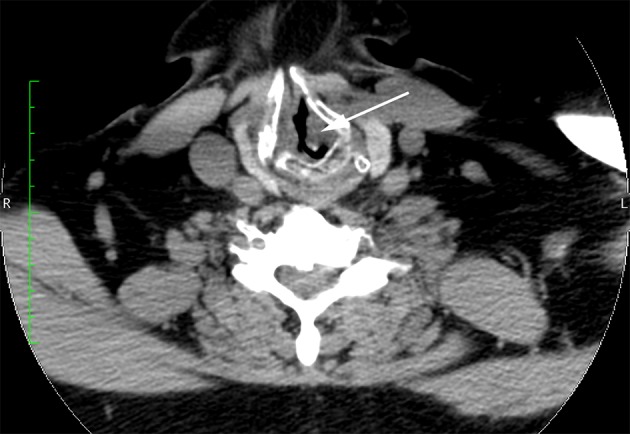
Enlarged Axial Computed Tomographic Scan Demonstrating Subglottic Stenosis (Arrow) and Narrowed Airway Lumen

Because of the patient’s symptoms and the authors’ videoendoscopy findings, the patient was referred to the otolaryngology clinic for further evaluation. Computed tomography revealed tracheal stenosis; a segment of the proximal subglottic trachea was narrowed to a diameter of approximately 4 mm (Figure 2). Computed tomography also showed cartilageous erosion consistent with tracheomalacia. The otolaryngology consultant initially proposed tracheal reconstruction, but this option was not feasible in the short term because of the urgent need to address the infected knee arthroplasty. The otolaryngologist concurred with the authors who were concerned that repetitive endotracheal intubation and extubation was not an option for this patient with a substantially narrowed glottis and subglottic stenosis. As a result, the patient underwent an elective permanent tracheostomy under conscious sedation (intravenous midazolam and fentanyl) to obtain definitive control of his airway immediately before the infected knee arthroplasty was removed and antibiotic spacers were placed after induction of general anesthesia. A subsequent incision and drainage procedure was also performed. The patient eventually underwent a revision total knee arthroplasty several months later after the infection had been successfully treated. The patient made an uneventful recovery after each operation.

## 3. Discussion

Anesthesiologists are well versed in the use of fiberoptic bronchoscopy for endotracheal intubation (11), but are less familiar with fiberoptic laryngoscopy or videoendoscopy as tools for preoperative airway evaluation, unlike their otolaryngology colleagues who routinely use these techniques (5).The safety and efficacy of transnasal flexible fiberoptic endoscopy has been well documented in patients with significant upper airway pathology including malignancy, tracheomalacia, and tracheal stenosis in the otolaryngology literature (5, 15). Rosenblatt et al. were the first anesthesiology group to describe preoperative endoscopic airway examination as a method to provide additional airway information in a study of 138 patients scheduled for elective upper airway surgery (14). These authors conducted their airway examinations on the day of surgery immediately before proceeding to the operating room and demonstrated that this strategy may provide specific new findings about airway anatomy. Their findings often altered the original plan for airway management, reduced the number of awake intubations, and identified patients in whom administration of neuromuscular blockers may be contraindicated because positive pressure ventilation or intubation may be unsuccessful (14). In contrast to Rosenblatt et al. who performed fiberoptic laryngoscopy shortly before surgery (14), the current authors have routinely performed this procedure as a tool for airway evaluation in the preoperative anesthesia clinic before anticipated elective surgery using dedicated high definition videoendoscopy equipment. As the current patient illustrates, the authors perform videoendoscopy through a transnasal approach in sitting position using topical anesthesia alone without the need for conscious sedation, very similar to the technique described for outpatient clinic airway evaluation by otolaryngologists (2, 4-6). After obtaining informed consent from the patient, the authors clearly document the specific indications for the procedure, the major clinical findings (including digital photographs taken with the videoendoscope), and any complications in the institution’s electronic medical record for subsequent use when the patient presents for surgery. This approach facilitates perioperative airway management planning well in advance of scheduled surgery and avoids potential delays associated with a fiberoptic or videoendoscopy examination performed immediately before the patient is transported to the operating room as previously reported (14).

The authors’ examination of the current patient demonstrated several important new findings, including a narrowed glottis, supraglottic scarring, subglottic stenosis, and redundant lower oropharyngeal tissue, suggesting that his airway may be difficult to repeatedly secure using conventional methods for his orthopedic surgery procedures. These observations, along with the computed tomography results, also indicated the possibility that proximal tracheal injury may occur with endotracheal intubation or that edema may compromise the patency of an already narrowed airway when the endotracheal tube was removed. As a result, the patient underwent an elective permanent tracheostomy to establish a definite airway for the anticipated operations required for treatment of his infected total knee arthroplasty. The authors did briefly consider performing a series of neuraxial anesthetics for the patient’s anticipated operations, but this option was dismissed because the patient had a long standing infection with the possibility of bacteremia despite chronic antibiotic therapy and also had a history of symptomatic lumbar spinal stenosis. The authors were also concerned that inadequate neuraxial or regional anesthesia during surgery may necessitate urgent airway intervention in this patient with significant airway abnormalities.

In summary, the current case illustrates that transnasal videoendoscopy conducted in the preoperative anesthesia clinic is capable of providing key information to guide airway management in patients with significant upper airway pathology.
